# Current Immunotherapy Approaches in Non-Hodgkin Lymphomas

**DOI:** 10.3390/vaccines8040708

**Published:** 2020-11-27

**Authors:** Robert Pytlik, Kamila Polgarova, Jana Karolova, Pavel Klener

**Affiliations:** 1 Institute of Haematology and Blood Transfusion, 128 00 Prague, Czech Republic; Robert.Pytlik@uhkt.cz; 2 First Department of Internal Medicine-Hematology, University General Hospital in Prague and First Faculty of Medicine, Charles University, U Nemocnice 2, 128 08 Prague 2, 110 00 Prague, Czech Republic; kamila.polgarova@vfn.cz (K.P.); jana.karolova@vfn.cz (J.K.); 3 Institute of Pathological Physiology, First Faculty of Medicine, Charles University, 128 53 Prague, Czech Republic

**Keywords:** non-Hodgkin lymphomas, CAR T-cells, bispecific antibodies, immune checkpoint inhibitors, immunomodulatory agents

## Abstract

Non-Hodgkin lymphomas (NHLs) are lymphoid malignancies of B- or T-cell origin. Despite great advances in treatment options and significant improvement of survival parameters, a large part of NHL patients either present with a chemotherapy-refractory disease or experience lymphoma relapse. Chemotherapy-based salvage therapy of relapsed/refractory NHL is, however, capable of re-inducing long-term remissions only in a minority of patients. Immunotherapy-based approaches, including bispecific antibodies, immune checkpoint inhibitors and genetically engineered T-cells carrying chimeric antigen receptors, single-agent or in combination with therapeutic monoclonal antibodies, immunomodulatory agents, chemotherapy or targeted agents demonstrated unprecedented clinical activity in heavily-pretreated patients with NHL, including chemotherapy-refractory cases with complex karyotype changes and other adverse prognostic factors. In this review, we recapitulate currently used immunotherapy modalities in NHL and discuss future perspectives of combinatorial immunotherapy strategies, including patient-tailored approaches.

## 1. Introduction

Non-Hodgkin’s lymphomas (NHL) comprise a group of more than 80 clinical entities according to the most recent WHO classification, which differ according to the cell of origin, clinical presentation, and prognosis [1]. While chemotherapy has been a mainstay of NHL treatment for more than 50 years, it was not equally effective in all NHL subtypes [2]. Immunotherapy has been successfully introduced to treatment of lymphomas more than 20 years ago, when a simple addition of anti-CD20 monoclonal antibody rituximab to combination chemotherapy significantly improved prognosis of almost all B-cell malignancies expressing this antigen [3]. However, a significant proportion of patients are either refractory or eventually relapse after the standard chemoimmunotherapy approaches. For these patients, new treatment modalities are urgently needed.

**Figure 1:**
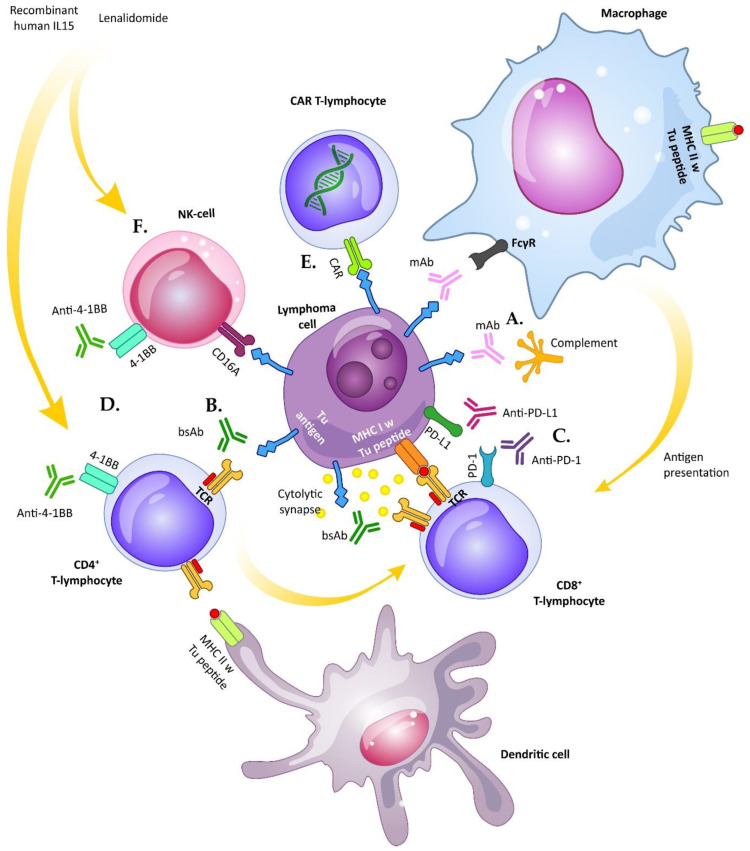
Overview of immunotherapy approaches in non-Hodgkin lymphomas. **Legend:** Simplified overview of basic principles of immunotherapy including monoclonal antibodies (**A**), bispecific antibodies (**B**), checkpoint inhibitors (PD-1, PD-L1) (**C**), activators of co-stimulatory molecules (4-1BB) (**D**), CARs (**E**), and immunomodulation (**F**). **Abbreviations:** bsAb = bispecific antibody; CAR = chimeric antigen receptor; FcγR = receptor for constant fragment (Fc) of immunoglobulin gamma; mAb = monoclonal antibody; MHC = major histocompatibility complex; NK = natural killer; PD-1 = programmed cell death 1; PD-L1 = PD-1 ligand 1; TCR = T-cell receptor; Tu = tumor.

The tumor microenvironment plays a critical role in lymphoma cell survival, growth, spread, and resistance to therapy. The landmark discovery that reactivation of tumor-suppressed cell-based immune responses can induce clinical remissions even in patients with chemotherapy refractory diseases led to the runaway development and testing of diverse new immunotherapy strategies and their combinations. Our better understanding of mechanisms of tumor immune escape and modes of action of anti-tumor immunity, together with unprecedented progress of genetic engineering enabled the concept and production of seemingly indefinite range of recombinant proteins and synthetic antibody constructs, including glycoengineered therapeutic monoclonal antibodies, bispecific antibody constructs, immune checkpoint inhibitors or activators, and genetically engineered T and NK cells carrying chimeric antigen receptors (CAR-T and CAR-NK cells) (Figure 1). In this review, we recapitulate the most relevant immunotherapy approaches for treatment of patients with non-Hodgkin lymphoma, both those routinely used and those in clinical development.

## 2. Monoclonal Antibodies

Monoclonal antibodies (mAbs) represent one of the most revolutionizing therapeutic approaches in the anti-cancer treatment. MAbs target a specific cancer antigen (Ag) eliciting a direct anti-tumor activity or evoking (an indirect) immunological response [4]. Other antibody-based approaches, such as antibody-drug conjugates and radioimmunoconjugates exert their mode-of-action predominantly by targeted delivery of the toxic payload to the lymphoma cells with little or no immunological effects and will not be discussed in this review. Each mAb molecule is formed by two antigen-binding fragments (Fab) at the N-terminal variable region (formed by V_L_ and V_H_) responsible for epitope recognition, and a constant fragment crystallizable (Fc) region at the C-terminus that mediates various immune responses [5]. These include recruitment of myeloid cells (e.g., macrophages, monocytes, mast cells etc.) and natural killer lymphocytes via Fcγ receptor (FcγR), which triggers antibody-dependent cell-mediated cytotoxicity (ADCC), antibody-dependent cell-mediated phagocytosis (ADCP) and activation of the classical complement cascade leading to formation of the membrane-attacking complex on the target cells (complement-dependent cytolysis, CDC) (Figure 1A) [6,7]. Some data also suggest that mAb treatment results in an adaptive anti-tumor effect [8]. Since the Fc region is responsible for the effector functions of mAbs, but possibly also for off-target toxicity, next-generation glycoengineered mAbs have been designed with targeted mutations of the Fc region in order to enhance their therapeutic efficacy and suppress toxicity. The Fc domain of immunoglobulin (Ig)G molecules is recognized by FcγR family of receptors including activating FcγRI, FcγRIIa, FcγRIIc and FcγRIII, and inhibitory FcγRIIb. The key surface receptor that mediates ADCC is FcγRIIIa, which can be found on the surface of natural killer (NK) cells, macrophages, monocytes, mast cells, eosinophils, and dendritic cells. In humans, two allotypes are present differing in a single amino acid (valine versus phenylalanine) at position 158, the first one presenting with higher affinity for Fc domain of IgG1. In contrast, ADCP activity of macrophages is mediated mainly by FcγRIIa (reviewed in [9]). Advances in genetic engineering enabled optimization of the interactions between Fc and FcγR during the design and production of next-generation mAbs for targeted enhancement of effectivity and reduction of side-effects. These techniques are based mainly on changes in the glycosylation profile of the antibody and on incorporation of amino acid mutations into the regions responsible for FcγRs binding. Point mutations of CH2 antibody domain may also be used to increase Fc binding to C1q thereby enhancing activation of the complement cascade [10]. In contrast to therapeutic monoclonal antibodies with enhanced ADCC/ADPC/CDC functions, targeted silencing of the Fc fragment-elicited immune effector function was employed to decrease toxicity of next-generation bispecific antibodies.

### 2.1. Rituximab and Next-Generation Anti-CD20 Antibodies

The first mAb approved in 1997 by United States Food and Drug Administration (US FDA) was rituximab—a chimeric human/mouse IgG1 anti-CD20 mAb. Rituximab soon became a standard of care for all CD20+ B-NHLs both in the front-line and salvage therapies (reviewed in [11]). Building on its unprecedented and lasting success, several biosimilars of rituximab have been adopted into clinical practice [12]. Since the start of the rituximab era, several other mAbs were evaluated in patients with NHL, but only a few of them reached clinical approval. Obinutuzumab (GA101) was the first Fc-glycoengineered anti-CD20 mAb with increased affinity for FcγRIIIa resulting in enhanced ADCC. Based on results of the clinical trials, obinutuzumab (in combination with chlorambucil) was approved for first-line treatment of patients with chronic lymphocytic leukemia (CLL) ineligible for fludarabine [13]. In the landmark GALLIUM and GADOLIN trials, the combination of obinutuzumab and chemotherapy prolonged the PFS in patients with follicular lymphoma (FL) compared to the combination of rituximab plus chemotherapy or to chemotherapy only both in the patients with refractory/relapsed (R/R) FL, and in the front-line setting [14,15]. Based on these data, obinutuzumab was approved for the treatment of FL patients in combination with chemotherapy. In diffuse large B-cell lymphoma (DLBCL), obinutuzumab in combination with cyclophosphamide, vincristine, doxorubicine, and prednisone (CHOP, G-CHOP) did not show superiority compared to Rituximab-CHOP (R-CHOP) standard of care [16]. Ofatumumab, another approved next-generation anti-CD20 mAb, has been approved for the therapy of CLL, but did not show clinical benefit in patients with NHL [17,18,19]. Ublituximab (TG-1101) is currently being evaluated in ongoing clinical trials with promising results in R/R B-NHL and CLL [20,21]. Other anti-CD20 antibodies (e.g., veltuzumab and ocrelizumab) did not reach wider clinical success [22,23].

### 2.2. Anti-CD19 Antibodies

The CD19 antigen represents another promising target extensively tested in experimental therapy of B-NHL. Tafasitamab (MOR-208, XmAb5574), a humanized anti-CD19 mAb with engineered Fc domain (leading to enhanced cytotoxic effect and decreased toxicity), is being evaluated in several ongoing clinical studies in patients with B-NHL [24]). Tafasitamab demonstrated clinical activity as a monotherapy in R/R B-NHL with overall response rate (ORR) 26–29% [25]. According to the results of a phase 2 clinical trial L-MIND, tafasitamab in combination with lenalidomide demonstrated promising clinical activity (ORR 60%, CR 43%) in patients with R/R DLBCL ineligible for high-dose therapy and autologous stem cell transplantation (HDT-ASCT), which led to its accelerated FDA approval in 2020 for the treatment of this target population [26]. Tafasitamab in combination with chemo(immuno)therapy is currently being assessed in the front-line therapy of patients with DLBCL (NCT04134936). A B-cell depleting humanized anti-CD19 antibody inebilizumab (MEDI-551), approved for immunosuppressive therapy of neuromyelitis optica, also showed activity in DLBCL and FL patients [27,28].

### 2.3. Anti-CD52 Alemtuzumab

The CD52 antigen is a glycopeptide present on both B- and T-lymphocytes. Alemtuzumab, a humanized anti-CD52 antibody was approved in 2007 for the treatment of R/R CLL patients. Alemtuzumab-based therapy is, however, complicated with high rates of infections, including life-threatening opportunistic infections due to prolonged T-cell depletion, which compromised its more widespread clinical use [29,30,31]. Alemtuzumab is recommended for the therapy of advanced stages of mycosis fungoides and Sézary syndrome and it remains the treatment of choice in patients with T-cell prolymphocytic leukemia either alone or in combination with purine analogues [32,33,34].

### 2.4. Anti-CCR4 Mogamulizumab

CC chemokine receptor 4 (CCR4) is highly expressed on regulatory T-cell subset (Tregs) and cutaneous lymphocyte antigen-positive skin-homing T cells including neoplastic cells of cutaneous T-cell lymphomas (CTCL) [35]. Anti-CCR4 antibody mogamulizumab showed clinical efficacy (ORR 50%, CR 28%, *n* = 28) in R/R T-NHL [36]. The promising results were then confirmed also in the upfront setting for adult T-cell leukemia/lymphoma, where it was combined with mLSG15 protocol with superior results in comparison to mLSG15 only (CR 52% resp. 33%) [37].

## 3. Bispecific Antibodies

**Figure 2:**
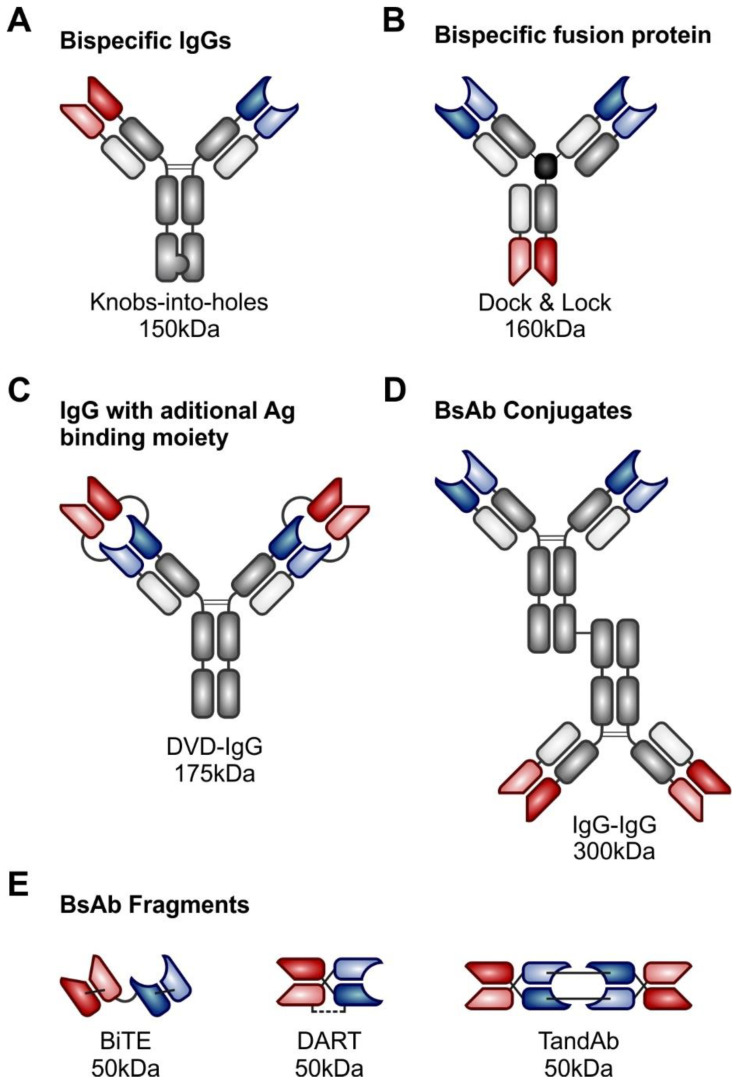
Alternative bispecific antibody formats in clinical development. **Legend:** (**A**) immunoglobulin gamma (IgG)-like bispecific antibody (bsAb); (**B**) bispecific fusion protein; (**C**) IgG with additional antigen binding moiety; (**D**) bsAb conjugates; (**E**) bsAb fragments including bispecific T-cell engagers (BiTE), dual-affinity re-targeting antibody (DART), and tandem diabody (TandAb). Dark and light colours represent heavy and light chains, respectively; blue and red colours represent variable fragments specific for different antigens.

Bispecific antibodies (bsAbs) are antibody-based molecules engineered to bind two different epitopes—one targets the malignant cells and the other one effector cells, usually T-lymphocytes, which mediate tumor cell destruction. Advances in bioengineering resulted in design and synthesis of a broad spectrum of bsAb formats with different cytotoxic activities in vitro and in vivo, different tissue penetration capabilities and different half-lives [38]. More than 100 currently known bsAb formats may be categorized into five different structural groups with particular construction issues: (a) bsAbs with IgG-like structure, with specific subtypes overcoming the light- and heavy-chain miss-pairing issues; (b) bsAbs with additional Ag binding unit; (c) bispecific fusion proteins with additional functionality or specificity, (d) bispecific antibody conjugates, which were more commonly produced before the recombinant methods were available, (e) bispecific antibody fragments with various approaches to connection of single chain fragment variable segments (scFv) (reviewed in [39,40]) (Figure 2).

Although the molecular details differ considerably, one of the main structural and functional categorizations can be based on the presence or absence of the Fc region. BsAbs lacking the Fc region are usually scFV-based constructs containing variable regions of heavy and light chains that are joined to each other by different approaches, such as non-immunogenic linker in bispecific T-cell engagers (BiTEs) or a hinge in case of dual affinity re-targeting antibodies (DARTs). These molecules are usually smaller, which may allow enhanced tissue penetration, but also results in very short half-life in vivo requiring continuous intravenous administration [40]. Bispecific antibodies with incorporated functional Fc region have longer half-life and can elicit ADCC and ADPC via activation of macrophages and NK cells (so called trifunctional antibodies) [41]. They may, however, hinder the cytolytic synapsis formation, lead to unwanted lysis of attracted T-cells, or increase the off-target toxicity, namely the cytokine release syndrome. As a consequence, some of the newer bsAb constructs (e.g., glofitamab or mosunetuzumab) contain targeted mutation(s) of the Fc binding sites, which mitigate these untoward effects [42].

### 3.1. T-cell Redirecting Bispecific Antibodies

As mentioned above, most of the discussed bsAbs are T-cell redirecting molecules. Currently, the most commonly targeted antigen on immune cells is CD3 on T-lymphocytes- such bispecific antibodies are called T-cell engaging bsAbs. The binding of CD3 on T-cells and tumor antigen expressed by leukemia/lymphoma cells allows recruitment and activation of T-cells with ensuing formation of the cytolytic synapse, release of perforin/granzyme B vesicles and targeted destruction of the malignant cells, which is virtually identical compared to cytolytic synapse triggered by antigen binding to T-cell receptor (Figure 1B) [43]. In order to avoid systemic activation of effector cells without the presence of target cells, the affinity of the Ab targeting CD3 is usually lower compared to the tumor Ag. Alternatively to CD3, CD5 binding can be used, but it was shown to be less potent in inducing T-cell activation comparing with CD3 stimulation [44]; anti-CD2 targeting did not lead to T-cell activation except for a co-stimulation with two anti-CD2 Abs. Curiously, the level of expression of CD20 did not correlate with anti-lymphoma efficacy of bsAb, but was dependent on the amount of T-cells [45].

### 3.2. First-Generation Bispecific Antibiodies: Blinatumomab

Blinatumomab was the first BiTE approved for the treatment of cancer, for patients with B-cell acute lymphoblastic leukemia (B-ALL). First-in-human studies for R/R B-NHL started around the year 2000 (MT103-1/01-2001, MT103-1/01-2002, MT103/01-2003), but did not show significant clinical efficacy (most probably due to inefficient dosing). These pivotal studies, however, revealed several pitfalls associated with the therapy based on BiTEs. The short in vivo half-life due to the structure of the molecule and its size of about 55 kDa required continuous intravenous infusion to maintain target plasma levels. Importantly, higher dosages led to unexpected adverse events including neurotoxicity and cytokine release syndrome (CRS) [46]. Subsequent studies with blinatumomab and other bsAbs therefore adopted a dose-escalating approach to ensure reaching the target dose with lower risk of complications [47]. For B-NHL, several dosages and dose steps were evaluated using 4 or 8 weeks continuous infusions. In a phase I study evaluating anti-lymphoma efficacy of blinatumomab in patients with R/R FL, DLBCL and mantle cell lymphoma (MCL), the maximum tolerable dose (MTD) was established as 60 μg/m^2^/day [47]. In a cohort of patients receiving MTD (*n* = 35), the overall response was 69%, including 55% for DLBCL patients. A phase 2 study of flat doses of blinatumomab in R/R DLBCL showed that the response was highly dependent on reaching the maximum dose [48]. In this dose escalation study (a flat dose of 9–28–112 μg/24 h), almost a third of patients did not reach the target dose due to rapid disease progression or due to toxicity, mainly neurological adverse events. For the evaluable subjects, the overall response was 43%, including 19% CR. Similar results were reported by a study enrolling 41 patients with aggressive NHL [49]. Altogether, 54% of patients discontinued the first cycle due to progression or toxicity, 32% had neurologic adverse event grade ≥ 3, and the ORR and CR was 37% and 22%, respectively. Blinatumomab is currently being evaluated in several prospective clinical trials in patients with R/R B-NHL, not only as a salvage therapy, but also as consolidation in DLBCL after autologous stem cell transplantation (Table 1).

### 3.3. Second-Generation Bispecific Antibodies

Second-generation bsAbs mosunetuzumab and glofitamab have been designed to improve the imperfections associated with the first generation bsAbs, namely with BiTE format blinatumomab. They have a silent Fc fragment that confers better pharmacokinetic properties with no need for continuous intravenous administration. In addition, better safety profile was achieved by silencing of the Fc fragment, by pretreatment with anti-CD20 obinutuzumab, and by ramp-up dosing during cycle 1, which all contribute to mitigation of cytokine release syndrome-associated toxicity. In addition to the beneficial combination of bispecific antibodies and anti-CD20 obinutuzumab, other combinations are currently being evaluated in diverse clinical trials (Table 2 and Table 3).

#### 3.3.1. Mosunetuzumab

Mosunetuzumab (RG7828, BTCT4465A), is a full length, fully humanized IgG1 bsAb targeting CD20 and CD3 with a modified Fc domain [50]. In preclinical studies, Fc silencing with resulting inactivation of ADCC did not compromise the cytotoxic efficacy of mosunetuzumab [45]. The interim results from a phase I/Ib clinical study in patients with R/R B-NHL (GO29781, Clinical Trials Identifier NCT02500407) demonstrated ORR and CR rates of 42.2% and 18.6% for aggressive B-NHL patients (*n* = 119) and 64.1% and 34.1% for indolent B-NHL (*n* = 64), respectively, with durable responses observed even in patients, who had failed prior CAR T-cell therapy [51]. The step-up dosing of mosunetuzumab during cycle 1 was employed to improve safety and enable safe escalation to higher, potentially more effective doses. Treatment-related AEs (CRS and neurotoxicity) were mild and majority occurred in cycle 1 [51,52]. Mosunetuzumab is currently tested in a several early phase clinical trials in patients with R/R B-NHL, both single-agent, and in combination with chemotherapy, antibody-drug conjugates, immune checkpoint inhibitors or lenalidomide (Table 2).

#### 3.3.2. Glofitamab

**Figure 3:**
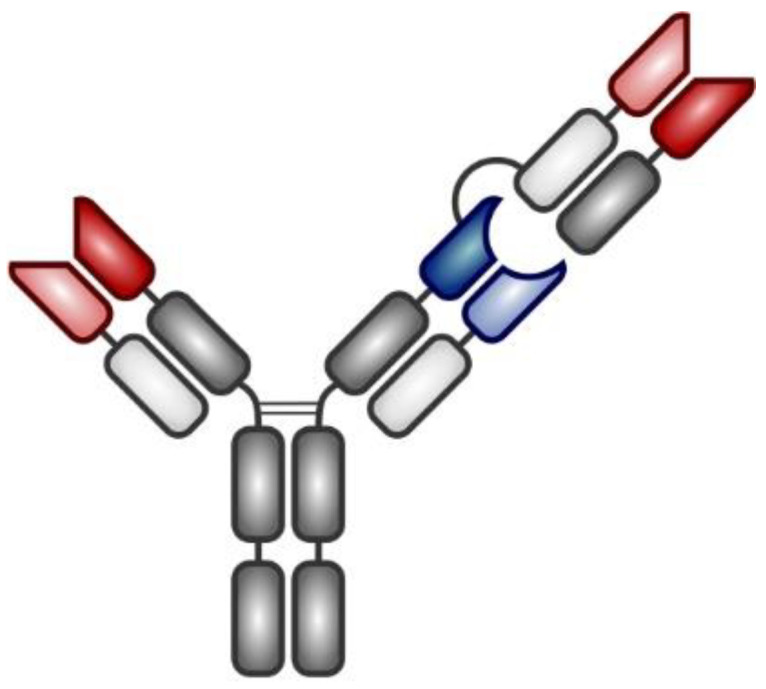
A simplified structure of glofitamab **Legend:** Blue and red colours represent variable fragments specific for CD3 and CD20, respectively.

Glofitamab (RG6026, RO7082859) is a bsAb targeting CD20 and CD3. Glofitamab contains three Fab regions (1 × CD3, 2 × CD20) and a silent Fc region (Figure 3).

Its higher affinity for CD20 enables glofitamab to compete for CD20 binding even at low doses with standard-of-care therapeutic anti-CD20 monoclonal antibodies rituximab or obinutuzumab [53,54]. Preliminary results from the first-in-human, multicenter, phase I, dose escalation study (NP30179, Clinical Trials Identifier NCT03075696) of glofitamab plus obinutuzumab in patients with R/R DLBCL, R/R primary mediastinal B-cell lymphoma (PMBCL), transformed (t)FL, and R/R FL were recently presented [55,56]. All patients received pretreatment with obinutuzumab 7 days prior to glofitamab based on preclinical data suggesting that obinutuzumab debulking would mitigate the risk of CRS-associated toxicity via decreasing the numbers of B-cells present in the peripheral blood. Within the 76 patients with aggressive R/R B-NHL (DLBCL, PMBCL, tFL) treated with different dose escalation protocols, the ORR and CR was 46% and 29%, respectively. In the cohort of patients with R/R FL, the ORR was 63% (50% CR). Toxicity was largely manageable with CRS grade ≥ 3 reported only in 5% of patients. Of note, severe neurotoxicity was not reported [55]. Median PFS in aggressive and indolent cohorts of patients was 2.9 and 14.9 months, respectively. Complete remissions were usually achieved early (after 3 cycles) and were durable. A phase 3 trial is currently evaluating anti-lymphoma efficacy of glofitamab versus rituximab in combination with gemcitabine and oxaliplatin in patients with R/R DLBCL (NCT04408638). Other trials are assessing combinations of glofitamab with PD-L1 inhibitor atezolizumab, ADC polatuzumab-vedotin, IMiD lenalidomide or CD19 targeted 4-1BB ligand RO7227166 (Table 3).

#### 3.3.3. REGN1979

REGN1979 is an anti-CD20 T-cell engaging IgG4-based bsAb with modified Fc domain. A phase 1 dose escalation study in R/R B-NHL (including 12 patients failing previous CAR19 T-cell therapy) did not show any dose limiting toxicities. Responses were dose-dependent with ORR ranging from 15% to 100%. Of note, lower doses of REGN1979 were needed for response induction in FL comparing to DLBCL patients [57]. Grade 3 CRS and neurotoxicity were reported in 7% and 3% patients, respectively, while grade 4–5 neurologic AEs were not observed. Selected ongoing clinical trials assessing novel bsAbs are shown in Table 4.

### 3.4. Natural Killer Cell-Activating Antibodies

Activation of natural killer (NK)-cells represents a relevant alternative to T-cell engagement. Several receptors capable of activating an immune response have been described. CD16A, FcγRIIIA, has been so far the most frequently used target as it triggers the activation without the need for any co-stimulatory signals [58]. Unfortunately, CD16 may be cleaved off the surface of NK cells by ADAM17 metalloproteinase [59]. This obstacle can be addressed by using ADAM17 inhibitor [60] or by targeting more activating receptors, such as NKp46 together with CD16A [61].

## 4. Immune Checkpoint Inhibitors

Immune escape is one of the crucial mechanisms responsible for tumor cell survival. Optimal anti-tumor T-cell responses require both presentation of immunogenic tumor antigens displayed on major histocompatibility complex (MHC) molecules and effective co-stimulation. In addition, lack of inhibitory signals from the tumor microenvironment (TME) normally associated with self-tolerance and T-cell exhaustion is indispensable for a sustained anti-tumor activity of effector T-cells. Any aberration in the process of antigen presentation, co-stimulation, or (lack of) T-cell inhibition may result in significant suppression of anti-tumor immunity. The recurrent aberrations observed in patients with NHL include lack of immunogenic tumor antigens, loss of expression of MHC molecules, interruption of co-stimulatory signals, and active tumor-induced immunosuppression [62]. Malignant cells are capable to shut-down anti-tumor T-cell responses by interactions with specific immune checkpoint molecules, the expression of which is associated with self-tolerance, T-cell dysfunction, and exhaustion [63]. Overexpression of lymphocyte activation gene-3 (LAG-3) on T-cells competes with CD4 for binding to peptide-MHC complex on antigen-presenting cells, thereby interfering with the antigen presentation process. Cytotoxic T-lymphocyte-associated antigen 4 (CTLA-4) binds to CD80 (B7-1) or CD86 (B7-2) and decreases activity of T cells by interrupting CD28-mediated co-stimulatory signaling. Aberrant overexpression of programmed death ligands 1 and 2 (PD-L1, PD-L2) or T-cell immunoglobulin and mucin domain 3 (TIM3) on malignant cells induce T-cell impairment, exhaustion, and apoptosis [64,65,66]. With the approval of several immune checkpoint inhibitors for the treatment of diverse solid cancers, it is now evident that the original proof-of-concept of inhibition of cancer-induced immunosuppression successfully translated into clinical practice. Since the first approval of ipilimumab for the treatment of melanoma in 2011, the new class of powerful anti-cancer immunotherapy agents dramatically changed the landscape of treatment options in clinical oncology and hematology [67]. In 2018, James P. Allison and Tasuku Honjo were awarded a Nobel Prize in Physiology and Medicine “for their discovery of cancer therapy by inhibition of negative immune regulation”. In hematologic malignancies, PD-1 blockade with nivolumab or pembrolizumab in R/R Hodgkin lymphoma became a flagship of immune checkpoint inhibition in lymphoproliferative disorders reaching ORR of 65% to 87% [66]. Curiously, in contrast to solid tumors and Hodgkin lymphoma, the implementation of checkpoint inhibitors into the treatment algorithms of NHL still remains a matter of investigation [68]. In this review, we focus primarily on the potential blockade of CTLA-4/PD-1/PD-L1/2 in experimental immunotherapy of NHL (Figure 1C). Table 5 displays selected checkpoint inhibitors currently evaluated for the therapy of NHL.

### 4.1. CTLA-4 Inhibition and Dual Immune Checkpoint Inhibition in NHL

Anti CTLA-4 antibody ipilimumab administered as a monotherapy in a phase 1 study in 18 patients with R/R B-NHLs was associated with only one complete remission in DLBCL cohort [69]. Dual checkpoint inhibition using ipilimumab in combination with nivolumab in R/R hematologic malignancies (CheckMate 039) resulted in only 20% ORR in B-NHL and 9% ORR in T-NHL cohorts [70].

### 4.2. PD-1, PD-L1/2 Inhibitors in Specific Subtypes of NHL

#### 4.2.1. Diffuse Large B-Cell Lymphoma

Diffuse large B-cell lymphomas (DLBCL) only poorly express PD-L1. Retrospective study of Kiyasu et al. performed on 1253 biopsies revealed that only 11% of DLBCL samples were PD-L1-positive (defined as ≥30% of neoplastic cells). The PD-L1 positivity was most frequently found in non-germinal center DLBCLs and in PMBCLs [71,72]. Single-agent inhibition of PD-1/PD-L1 was largely ineffective in salvage therapy of R/R DLBCL. Similarly, a nivolumab-based consolidation in R/R DLBCL patients ineligible for HDT-ASCT was associated with very low response rate (NCT02038933) [73]. In contrast, PD-1 blockade with pidilizumab showed clinical benefit in patients with DLBCL after HDT-ASCT suggesting a potentially new therapeutic strategy [74]. Another phase II study using PD-1 blockade as a maintenance in high-risk B-NHLs has only recently been initiated (NCT03569696). Besides single-agent approaches, several combinatorial treatment regimen incorporating PD-1/PD-L1 inhibitors are currently being investigated in ongoing clinical trials in DLBCL. The combination of nivolumab with R-CHOP is currently being assessed in a phase I/II study in patients with so far untreated B-NHLs (NCT03704714). A phase II study of anti-PD-L1 antibody durvalumab, either in combination with R-CHOP (arm A) or with lenalidomide and R-CHOP (arm B) has been recruiting patients with previously untreated high-risk DLBCL (NCT03003520).

#### 4.2.2. B-NHL with Recurrent Gains of 9p24.1

Amplification or copy number gains of 9p24.1 have been associated with increased expression of PD-L1 and PD-L2 ligands. In addition to Hodgkin lymphoma, such genetic aberrations have been recurrently reported in patients with PMBCL, primary central nervous system lymphoma (PCNSL), mediastinal gray zone lymphoma (MGZL), and primary testicular lymphoma (PTL) [75,76,77,78]. PMBCL accounts for approx. 10% of DLBCL and has some overlapping features with Hodgkin lymphoma. PCNSL and PTL are rare subtypes of B-NHL with shared genetic features. The prognosis of patients with R/R PMBCL, PCNSL, or PTL remains dismal. Increased expression of PD-1 ligands suggested that these NHL subtypes might be susceptible to PD-1 inhibition. Indeed, anti-PD-1 pembrolizumab was active in patients with R/R PMBCLs in two landmark clinical trials Keynote-013 (NCT01953692) and Keynote-170 (NCT02576990) with ORR 45% and 48%, respectively [79,80]. At 12 months, PFS was 47% and 38%, respectively. In a phase 2 CheckMate 436 trial (NCT02581631), 30 patients with R/R PMBCL treated with the combination of another PD-1 inhibitor nivolumab with anti-CD30 antibody-drug conjugate brentuximab vedotin had 73% ORR with 37% CRs [81]. Nivolumab also showed clinical benefit in a small cohort of five patients with R/R PCNSL and PTL with 4 patients achieving CR and 1 patient PR [76]. Clinical activity of nivolumab in patients with R/R PCNSL and PTL is currently assessed in an ongoing trial CheckMate 647 (GovTrial Identifier NCT02857426).

#### 4.2.3. EBV-Positive NHL

In addition to genetic lesions, Epstein-Barr virus (EBV) infection was reported to induce PD-L1 expression [82]. Expression of PD-L1 was confirmed in more than 90% EBV-positive tumor cells. It was also reported that EBV-positive NHLs have higher PD-L1 expression (56%) when compared to EBV-negative NHLs (11%) [83,84]. Pembrolizumab was also investigated in patients with R/R NK/T-cell lymphomas with 5 out of 7 patients achieving CR. These remarkable observations could be explained by EBV-driven increase of PD-L1 expression in NK/T-cell lymphomas [85,86]. Overall, the immune-checkpoint inhibition seems to be a relevant option for the treatment of EBV+ lymphomas.

#### 4.2.4. Indolent Lymphomas

Follicular lymphoma (FL) is an indolent lymphoma with abundant levels of PD-1-positive infiltrating T-cells [87]. Despite that, single-agent PD-1 blockade with nivolumab or pembroliuzmab was associated with very limited activity in patients with R/R FL suggesting that combinatorial approaches might be more beneficial in this type of NHL [88,89]. Indeed, PD-1 blockade with pidilizumab in combination with rituximab was active in patients with R/R FL resulting in 66% ORR [90,91]. In a study of 25 patients with relapsed/refractory chronic lymphocytic leukemia (R/R CLL) including 9 patients with Richter transformation, the objective responses were observed exclusively in the patients with Richter transformation (44% ORR compared to 0% ORR in CLL) [92].

#### 4.2.5. T-Cell Lymphomas

T-cell lymphomas (TCL) express PD-1 and PD-L1 molecules on both tumor cells and non-malignant T lymphocytes, which may result in a profoundly immunosuppressive environment [93]. Recently published results from a CITN-10 phase 2 trial (NCT02243579) demonstrated significant antitumor activity with durable responses in patients with advanced cutaneous T-cell lymphomas (CTCL, mycosis fungoides, and Sézary syndrome) [94]. Clinical studies assessing combined treatment in peripheral T-cell lymphomas, such as nivolumab in combination with chemotherapy EPOCH (NCT03586999), or pembrolizumab in combination with histon deacetylase inhibitor romidepsin (NCT03278782) are currently recruiting patients. Preliminary results of the pembrolizumab and romidepsin combination had ORR 44% [95].

### 4.3. 4-1BB/CD137 Activators and Gain of Effector Functions

Ligation of a co-stimulatory molecule 4-1BB/CD137 present on T lymphocytes with its ligand (4-1BBL) improves T-cell activation and promotes survival and inhibition of activation-induced cell death (Figure 1D). Besides T-cells, 4-1BB is also expressed on dendritic cells, NK-cells, mastocytes or eosinophils with their possible activation after its stimulation. Anti-CD137 agonistic antibody utomilumab is a fully-human monoclonal antibody able to activate NF-kB signalization, and foster human leukocyte proliferation and anti-tumor immune responses [96]. A phase I study of utomilumab in combination with rituximab in R/R CD20-positive NHL had ORR 21.2% (NCT01307267) [97]. Utomilumab is currently being evaluated in a phase 1b/3 study in combination with anti-PD-L1 avelumab, anti-CD20 rituximab and conventional chemotherapy in R/R DLBCL (NCT02951156). Bispecific Abs targeting a tumor-antigen and CD137 demonstrated clinical activity in combination with T-cell engaging bsAbs in diverse solid and hematological malignancies [98,99].

### 4.4. Innate Immune Checkpoint Blockade in NHL

Overexpression of CD47 or its ligand SIRPα transmits a “do not eat me“signal to all macrophages and monocytes, thereby suppressing antibody-dependent cell-mediated phagocytosis. Consequently, disruption of CD47-SIRPα signaling represents a mechanism of immune escape for many malignant cells [100,101,102]. A phase 1 trial using anti-CD47 antibody Hu5F9-G4 in combination with anti-CD20 rituximab enrolled heavily pretreated patients with DLBCL (15/22) and FL (7/22). The ORR in DLBCL and FL reached 40% and 71%, respectively, confirming a clinical benefit in the heavily pretreated NHL patients [103]. A SIRPα-immunoglobulin G1 Fc fusion protein TTI-621 was active in early-stage trials in patients with R/R DLBCLs as well as R/R CTCL [104,105]. Combination of anti-CD47 antibody magrolimab with anti-CD20 rituximab in R/R B-NHL (NCT02953509) as well as combination therapy using TTI-622 (another SIRPα-immunoglobulin G4 Fc fusion protein) in experimental therapy of R/R lymphomas and myelomas is currently recruiting patients (NCT03530683). Selected studies incorporating immune checkpoint inhibitors in experimental therapy of NHLs are displayed in Table 6.

## 5. Chimeric Antigen Receptor-Based Adoptive Immunotherapy

Chimeric antigen receptor (CAR)-based therapy differs from other anticancer therapies both by the mechanism of action and from the manufacturing and regulatory perspective (Figure 1E). CAR-engineered cells are included among advanced therapy medicinal products (ATMP) according to both EMA and US FDA and despite being living cells, they are legally covered by Pharmaceutical Acts, in contrary to Cells and Tissues Acts, which regulate products for hematopoietic cell transplantation [106]. The manufacturing of CAR engineered cells must follow both the good manufacturing practice (GMP) principles (as stated e.g., by EudraLex), and regulations for genetically modified cells. However, because of recent advances in fully automated closed system manufacturing (e.g., on clinical-scale magnetic-assisted cell sorting (CliniMACS) Prodigy platform), these cells may be prepared conveniently virtually at the bedside [107,108,109]. In addition, after product registration, CAR engineered cells may be cryostored together with non-modified cells and tissue products. From the clinical point of view, attractiveness of CAR-based products stems from the fact that it is apparently effective in patients refractory to both conventional and high-dose chemo(immuno)therapy and even in cases of immune escape after allogeneic stem cell transplantation. Furthermore, CAR-based therapy in these days usually comprises a single infusion of the engineered lymphocytes, making this treatment generally well tolerated, though it is associated with some unique toxicities. Currently marketed products are all T-cell based, manufactured by transduction of autologous cells obtained by unstimulated leukapheresis, and all are directed against CD19 antigen. In vivo expansion of infused T-cells is facilitated by pre-infusion lymphodepleting chemotherapy, usually fludarabine and cyclophosphamide. First reports about successful construction of fusion receptors were published in 1991 by three independent teams, which coupled CD3ζ to the CD4, CD8 or CD25 extracellular domains [110,111,112]. However, it took more than 25 years for the approval of the first CAR-based therapy tisagenlecleucel (tisa-cel, Kymriah^®^, Novartis, Basel, Switzerland) by US FDA in August, 2017 for treatment of R/R B-ALL and DLBCL. Axicabtagene ciloleucel (Axi-cel, Yescarta^®^, Gilead/Kite), the second CAR19 T cell product, was approved in October 2017 for the therapy of R/R DLBCL and brexucabtagene autoleucel (Tecartus^®^ Gilead/Kite) in July 2020 for therapy of R/R MCL. Lisocabtagene ciloleucel (liso-cel, Celgene/Juno) is currently being evaluated in large prospective clinical trials in patients with R/R DLBCL and is under review process by both FDA and EMA regulatory authorities.

### 5.1. Basic Principles of CAR Design

**Figure 4:**
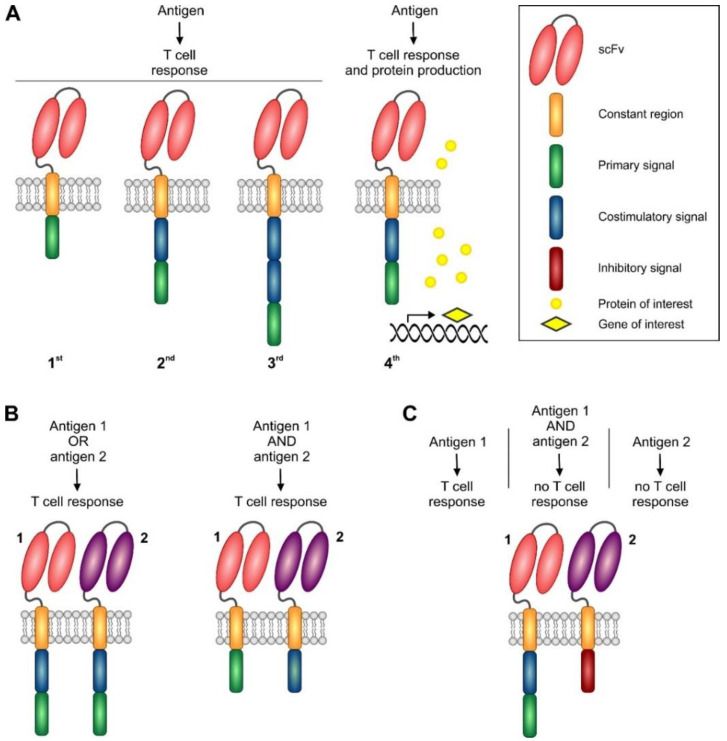
Schematic representation of design of four generations of CAR. **Legend: Conventional CARs:** (**A**) 1st to 3rd generation are defined by their signaling domains: a primary signaling domain only (1st generation); signaling and co-stimulatory domains (2nd generation); combined co-stimulatory domains (3rd generation); a release of activating cytokine upon CAR engagement (4th generation). **Co-expression of two different CARs:** (**B**) engagement of either CAR triggers downstream activation (left); engagement of both CARs triggers downstream activation (right); (**C**) engagement of inhibitory CAR prevents T-cell activation in the presence of cells that express the target antigen 2.

Chimeric antigen receptor engineering is a complex procedure, which was recently covered in several excellent reviews [113,114,115,116]. Basically, all CARs contain an extracellular ligand-binding domain, a spacer domain, a transmembrane domain, and cytoplasmic domains (Figure 4).

The extracellular binding domain is most commonly a single-chain fragment of the variable region of an antibody (scFv), where heavy and light chain variable regions (V_H_ and V_L_) are connected by a short (glycine-serine) peptide sequence. However, scFvs are prone to misfolding, which results in loss of affinity to the targeted antigen. Two non-covalently bound polypeptide chains were therefore used as an alternative [117]. Nanobodies are single-domain antibody fragments consisting of heavy-chain-only variable regions of antibodies, which have affinities similar to scFvs, but are less prone to misfolding [118,119]. Other than antibody-derived binding domains can be used as well. For example, a natural killer group 2 member D receptor (NKG2D) is a C-type lectin-like activating immune receptor present on NK cells. It binds to „stress ligands“ including MHC class 1 chain-related proteins A and B (MICA, MICB) and cytomegalovirus UL16 binding proteins (ULBP1-6) [120], which are upregulated on tumor cells. NKG2D receptor can be introduced via CAR both into T-cells, and into NK cells, because in unmanipulated NK cells NKG2D is downregulated by tumor microenvironment [121]. Combination of NKG2D with several co-stimulating domains, as DAP-10, 4-1BB, CD3ζ, and CD28 further enhanced NK cells inherent cytotoxicity [122]. Yet another approach to possibly universal CAR constructs is streptavidin as a biotin-binding domain, which was recently employed by Lohmueller [123]. This approach may have its added value in facilitating selection and in vivo detection of CAR-T cells.

#### 5.1.1. CAR Spacer and Transmembrane Domains

The spacer domain connects the extracellular binding domain to the transmembrane domain. As effective immune synapse requires approximately 15 nm distance between target and immune cells, the length of spacer domain should be tailored according to epitope distance from cancer cell surface [113]. Besides the length, various other problems can be encountered with different linkers. IgG1 constant domain-based linker can bind to FcγRI, which may cause off-target activation of macrophages leading to lysis of CAR cells. IgG4 hinge region, used in liso-cel, elicits weaker FcγRI binding. Further engineering of IgG4 hinge may also lead to increased receptor dimerization and increased cytotoxicity [124]. The CD28 hinge region, used in axi-cel, and CD8 hinge used in tisa-cel do not induce FcγR binding. Of these two, CD28 spacer is longer, which may lead to increased activation-induced cell death (AICD) [125]. However, a study comparing CD28 and CD8 spacers used different CAR construct than that used in the currently available products. Therefore, differences in axi-cel and tisa-cel properties are probably caused by other factors in CAR design. The transmembrane domain may cause receptor-receptor interactions, which may result in CAR dimerization, but also interactions between CAR and T-cell receptor (TCR), when a native CD3ζ transmembrane domain is induced in the construct. The resulting canonical T-cell signaling can contribute to CAR T-cell activation [126].

#### 5.1.2. First and Second Generation CARs

Intracellular signaling domains are responsible for most of the differences observed in the currently manufactured CAR T-cells and therefore, are of particular research interest. According to the number and type of intracellular domains, CAR constructs may be divided into several generations. First generation of CARs utilized only one stimulatory signal (“signal 1”), usually by CD3ζ or FcεRIγ [110,111,112,127]. CAR-T cells with these constructs proliferated poorly and were prone to AICD. Addition of another stimulatory signal (“signal 2”), or a co-stimulatory signal, led to second generation CARs, which are represented by all three currently approved or soon-to-be approved products. While axi-cel uses CD28 co-stimulation, both tisa-cel and liso-cel use 4-1BB domain. CD28 confers faster and more robust intensity of signaling compared to 4-1BB, which results in more rapid expansion of T-cells, but also in their shorter persistence in vivo due to differentiation to the effector memory phenotype [128,129]. In addition, CD28 CAR engineered cells are more prone to tonic signaling and AICD [130]. Based on these results, it was assumed that CD28 co-stimulation leads to faster expansion and tumor eradication, while 4-1BB co-stimulation may lead to longer persistence and better long-term protection. However, a recently published clinical study, which co-infused CD28 and 4-1BB CAR-T engineered cells, did not allow for such simple conclusion, because of high inter-individual variations of expansion patterns of both cell types [131]. Currently, it appears that both CD28 and 4-1BB-engineered CAR-T products have similar therapeutic efficacy, and that more severe acute toxicities of axi-cel are probably related to its faster in vivo expansion. Of other co-stimulatory domains, an inducible T-cell co-stimulator molecule (ICOS) may have theoretically some advantages over CD28 and 4-1BB. ICOS-based co-stimulation led to TH1 and TH17 polarization, while TH17 CAR-T cells with ICOS co-stimulation showed longer persistence in experimental mice than those with CD28 or CD4-1BB [132].

#### 5.1.3. Third Generation CARs

Third generation CARs employ, generally, two co-stimulatory molecules, though in principle, even more co-stimulatory domains may be added to the construct [122]. Co-stimulation with CD28 and 4-1BB in 3rd generation CAR T-cells led to increased phosphorylation of signaling proteins in comparison to CD28-only co-stimulated 2nd generation CAR T-cells, which resulted in better expansion in vitro and in experimental animals [133]. Correspondingly, 3rd generation CAR T-cells showed faster expansion and longer persistence than 2nd generation CAR T-cells when simultaneously applied to patients with B-cell malignancies, especially when the tumor burden was low [134]. However, clinical relevance of these findings has to be confirmed in prospective clinical trials. ICOS and 4-1BB co-stimulation was also used in 3rd generation CAR T-cells, generally with similar results to CD28 and 4-1BB dual co-stimulation, though with a potentially different expansion kinetics in 3rd generation CD4+ CAR-T cells compared to CD8+ cells [135]. On the other hand, when CD28 and OX40 co-stimulation was employed in cytokine-induced killer cells (a subset of activated T-cells co-expressing NK-cell markers), it resulted in increased AICD and shorter persistence of cells in comparison to those co-stimulated with CD28 only [136]. Therefore, more is not necessarily better, and the result probably depends both on co-stimulatory molecules and on the type of the transduced cells.

#### 5.1.4. Next Generation CARs and Future Approaches

CAR constructs utilizing other concepts than multiple co-stimulation are generally called next-generation CARs. Sometimes, terms “fourth generation CARs” or T-cells redirected for universal cytokine-mediated killing (TRUCKs) are used to define constructs that employ an expression cassette for a transgenic cytokine [137]. Either a constitutive or activation-induced protein synthesis may be employed. In interleukine 12 (IL-12)-equipped TRUCKs, the induced cytokine expression not only acted in an autocrine fashion, but also led to recruitment and activation of innate immune cells [138]. IL-15 is necessary for expansion of NK cells, and equipment of CAR-NKs with this cytokine expression system successfully enhanced their otherwise limited persistence and expansion in vivo [139]. Another strategy of next-generation CAR engineering involves transduction of cells by two different CARs, both equipped with co-stimulation domains, leading to elimination of lymphoma cells, on which just one of the two antigens of interest is expressed. Alternatively, one CAR can harbor an inhibitory domain, which would prevent immune response if both antigens are present on the recognized cell. The second concept can be employed for targeted elimination of malignant cells with aberrant loss of expression of a physiological antigen [113].

### 5.2. Clinical Results with Registered CAR Products

Currently, there are no published results from phase III clinical trials comparing CAR-based therapies with other treatment modalities. Results of registration studies for R/R aggressive B-NHL after failure of at least two previous lines of therapy with currently or soon-to-be marketed CAR T-cell products are summarized in Table 7 [140,141,142,143].

The registration studies included predominantly patients with R/R DLBCL, but also with other subtypes of aggressive B-NHL including PMBCL, transformed aggressive lymphomas, double or triple-hit lymphomas. Comparison of treatment results is somewhat difficult, because of different relative proportions of particular diagnoses, differences in treatment schedules, and reporting of outcomes. Current consensus is that from efficacy standpoint, these products are roughly equivalent. More toxicities associated with axi-cel may be attributed to the CD28 co-stimulatory domain used in this product. Different grading criteria for CRS may also contribute, as the Penn grading system results in higher number of grade 3–4 CRS in comparison to Lee system [144,145]. Real-world data confirmed results of the pivotal studies in general patient populations [146]. Analyses focused on older fragile patients and patients with CNS involvement reported safety and efficacy also in these clinical situations [147,148]. Brexucabtagene autoleucel (formerly KTE-X19) differs from its related product, axi-cel, by a production step involving depletion of potentially malignant CD19 cells from the source material. In MCL patients refractory both to anti-CD20 rituximab and Bruton tyrosine-kinase (BTK) inhibitors (ibrutinib, acalabrutinib), 67% of patients achieved CR (93% ORR), and PFS was 61% at 12 months, which is unprecedented in this patient population. Of note, the efficacy was independent of all currently known adverse prognostic markers including complex cytogenetic changes, blastoid morphology, and high proliferation index by Ki-67. CRS frequency and severity was similar to axi-cel in DLBCL, with more frequent neurotoxicity (63%, with 32% grade 3–4) [149].

### 5.3. Future Directions

Several pipelines of CAR-based strategies are currently being pursued. Selected ongoing clinical trials evaluating CAR-based therapies in patients with diverse lymphoid malignancies are displayed in Table 8.

#### 5.3.1. CAR T-Cells in Low-Grade Lymphomas

Efficacy of CAR T-cells in low-grade lymphomas is the main focus of the ZUMA-5 trial (NCT03105336), where axi-cel showed 95% ORR in follicular lymphoma and 81% in marginal zone lymphoma, with CR rates 81% and 75% and median duration of response 20.8 and 10.6 months, respectively [150]. More data are necessary to evaluate these results in the context of numerous other treatments currently available for these diagnoses. In CLL/SLL, the success of CAR T-cell therapy has been relatively modest so far [151]. T-cells from CLL patients have increased expression of exhaustion markers CD244, CD160, and PD-1 [152]. KTE-X19 is currently being evaluated for patients with R/R CLL in a phase 1 clinical trial (NCT03624036). Removal of tumor cells from the apheretic material during KTE-X19 production reduces the possible activation and exhaustion of CAR19 T-cells during the ex vivo manufacturing process. Another viable strategy in CLL/SLL patients is to combine CAR T-cells with BTK inhibitors (ibrutinib, acalabrutinib, zanubrutinib). BTK is a key component of the signaling pathway of B-cell receptor (BCR) and its inhibition by ibrutinib is very effective in CLL/SLL [153]. Patients treated with ibrutinib for at least one year before CAR T-cell therapy had fewer T-cell exhaustion markers, a better profile of CAR T-cell expansion, and an improved clinical outcome [154]. Furthermore, ibrutinib reduces IL-6, IFNγ, TNF-α, and GM-CSF and may be also effective in CRS prevention [155]. A phase 1 clinical trial of tisa-cel with ibrutinib for CLL/SLL is currently recruiting patients (NCT03331198). In addition, administration of another BTK inhibitor, acalabrutinib, before leukapheresis, is currently being evaluated (NCT04257578).

#### 5.3.2. CAR T-Cells in T-NHL

Most targetable antigens in patients with T-NHL (e.g., CD2, CD5, CD7) are expressed both on non-malignant and neoplastic T-cells. Consequently, they would be shared also by the manufactured CAR T-cells, which would inevitably result in fratricide killing. It is also technically difficult to deplete malignant T-cells from the apheretic material. CD30 is an antigen present exclusively on malignant lymphocytes in patients with Hodgkin lymphoma and some T-NHL subtypes (especially on anaplastic large T-cell lymphomas). However, while results of two parallel phase 1/2 studies (NCT02690545 and NCT02917083) evaluating efficacy of CAR30 T-cells were recently reported in the cohort of patients with Hodgkin lymphoma, results of the cohort of CD30+ NHL were not published so far [156]. Another experimental approach is engineering of CAR T-cells against constant portions of T-cell receptor (TCR). During TCRβ chain rearrangement, mutually exclusive selection of either TRBC1 or TRBC2 occurs. Targeting the constant region chosen by cancer cells spares significant numbers of physiological cells and also eliminates fratricide killing [157]. A phase 1/2 study of AUTO4, anti-TRBC1 CAR T-cells for R/R T-NHL is ongoing (NCT03590574). CD4CAR product uses only CD8+ cells for manufacturing, which also overcomes the fratricide killing and reduces probability of the tumor cell transduction [158]. A phase 1 study for R/R CD4+ T-NHL is currently recruiting patients (NCT03829540).

#### 5.3.3. CAR T-Cells as a Consolidation Therapy

Salvage therapy and HDT-ASCT consolidation remains the current standard of care for all patients with chemotherapy-sensitive high-grade NHL at the first clinical relapse. However, only one quarter of these patients eventually reach the transplantation, the major obstacles being advanced age, general frailty, or chemoresistance. A phase 2 TRANSCEND-PILOT-017006 (NCT03483103) trial is assessing efficacy of liso-cel as consolidation for patients not eligible for HDT-ASCT because of age or comorbidities. A ZUMA-12 trial (NCT03761056) offers CAR T-cell consolidation for patients with persistent PET positivity after 2 cycles of standard first-line therapy. Three different phase 3 trials are currently randomizing patients between HDT-ASCT and CAR T-cell-based therapy (NCT03391466, NCT03575351 and NCT03570892). Consolidation of remission after HDT-ASCT was studied with axi-cel; however, the toxicity was high, and 2-year PFS reached only 30% [159]. A similar approach is adopted in T-NHL patients after HDT-ASCT with anti-CD30 CAR T-cells (NCT02663297). Donor-derived anti-CD19 CAR T-cells as a consolidation therapy after T-cell depleted allogeneic stem cell transplantation is another strategy currently being assessed in a phase 1 trial (NCT04556266).

### 5.4. Mechanisms of Resistance to CAR T-Cells

More than one half of the patients treated with diverse CAR19 T-cells are either refractory or relapse after the achievement of remission. Though the loss of CD19, which is a common cause of CAR19 T-cell resistance in B-ALL, appears uncommon in B-NHL, CD19 mutations were reported in CAR19 T-cell refractory patients [160]. All currently marketed CAR T-cell-based products use the same scFv construct (FMC63), the most plausible reason, why retreatment with alternative CAR19 T-cell product was associated with limited clinical responses [161]. Theoretically, CAR construct against a different CD19 epitope might be used; however, from the practical point of view, it is reasonable to use CAR against a different antigen. CAR T-cells recognizing CD20 antigen are currently being evaluated in several clinical trials, single-agent (NCT03664635) or in combination with CAR T-cells with distinct specificity (NCT03125577) [162]. Advances in CAR T-cell engineering also allow for the preparation of bispecific or multispecific CAR T-cell products (NCT04186520).

The tumor microenvironment may also contribute to CAR T-cell resistance. Combination studies of CAR T-cells with immune checkpoint inhibitors and/or small immunomodulatory drugs that should overcome the tumor-induced CAR T-cell suppression are ongoing (NCT03310619 PLATFORM, NCT 03,287,817 ALEXANDER). Besides the combinations with diverse anti-PD-1 and anti-PD-L1 antibodies, CAR T-cells may be engineered to possess a chimeric PD-1-CD28 gene, which mediates stimulation of the cells upon PD-1 engagement (NCT04163302) [163]. Because advanced genetic manipulation of CAR T-cells may theoretically result in serious systemic toxicities, safety switches are being introduced into CAR constructs. A representative example of the safety switch is inducible caspase 9 (iC9), which may be activated by rapamycin or rimiducid (NCT03016377) [164].

### 5.5. Universal CAR T-Cells

Only autologous CAR T-cells are currently available for clinical use. However, custom-made manufacturing of autologous CAR T-cells is quite difficult from both clinical and logistic point of view. First, patients after multiple lines of treatments may not have enough T-cells for successful apheresis, or these cells may be of inferior quality. Second, the manufacturing procedure takes time, which may be critical especially for patients with biologically aggressive lymphomas. Though in theory, a CAR T-cell product may be available in two weeks after collection of starting material; current reality (at least in Europe) is around four weeks. Universal “off-the-shelf” CAR T cell (UCART) concept is therefore an attractive strategy. However, currently employed CAR T-cells have preserved native TCR receptors on their surface and consequently can elicit graft-versus-host disease (GvHD). Manufacturing of UCARTs thus requires additional engineering. T-cell receptor, or part of it, may be deleted by transcription-activator-like effector nucleases (TALEN) or CRISPR/Cas9 technologies, though currently employed protocols resulted only in partial TCR deletion and resulting products may still carry the risk of GvHD. Cooper et al. developed a CRISPR/Cas9 edited anti-CD7 T-cells, where both CD7 and TCRα are deleted [165]. This product has successfully undergone preclinical evaluation and a phase I clinical trial is being planned. A related autologous product, anti-CD7 CAR T-cells with deleted CD7, is already being tested in a phase 1 clinical trial in patients with T-cell malignancies (NCT03690011, CRIMSON).

### 5.6. CAR-Engineered NK Cells

NK cells, in contrast to cytotoxic T lymphocytes, kill target cells in HLA-unrestricted manner and therefore do not elicit GvHD. NK cells recognize all class I HLA antigen-expressing tissues as “self” and kill only those tumor cells that lack them. CAR expression in NK cells results in targeted killing with theoretically minimal side effects. CAR design for NK cells possess different challenges compared to T-cell engineering. The viral transduction systems used for T-cells achieved less than 5% transduction in case of NK cells [166]. Transduction efficacy may be improved by drug-induced inhibition of intracellular innate immune receptors in NK cells, or non-viral transduction systems based on electroporation or nucleofection may be used [167,168]. Another peculiar problem with peripheral NK cells is their insufficient in vivo proliferation. In theory, allogeneic NK cells might be administered repeatedly, but this can result in alloimunization and subsequent elimination of CAR-NK by host immune system. IL-15 containing cassettes may solve the problems with insufficient expansion [139]. Instead of peripheral NK cells, cord blood cells, NK-92 cell line, or induced pluripotent cells may be used for the manufacturing of CAR NK cell product [139,169,170]. As IL-15 is potentially leukemogenic, the NK cells were also equipped with the inducible suicide caspase 9 gene to enable their rapid elimination in case of uncontrolled proliferation; however, no such serious adverse event has been reported so far in a clinical trial of allogeneic cord blood-derived CAR NK cells [171]. Several clinical trials with various CAR-engineered NK cells are being evaluated in ongoing clinical trials (NCT03056339, NCT03774654) [172].

## 6. Immunomodulatory Agents (IMiDs)

Immunomodulatory agents are small molecule cereblon-modulating chemical agents that have both direct anti-tumor effects and indirect (anti-angiogenic and immunomodulatory) activities [173]. Besides the prototypical agent lenalidomide, other IMiDs are currently under clinical investigation including CC-122 avadomide, CC-200 iberdomide, and others. Besides their single-agent anti-tumor activites, IMiDs are more and more frequently used as part of combinatorial treatment approaches including combinatorial immunotherapy strategies, e.g., in combination with monoclonal antibodies, checkpoint inhibitors, bispecific antibodies or CAR T-cells (Table 9).

### 6.1. Lenalidomide

Lenalidomide, a thalidomide derivative, is an orally available IMiD used for the treatment of multiple myeloma and several types of B-NHL. Its mechanisms of action are both direct-mediated through stimulation of E3 ubiquitin-ligase cereblon, and indirect-mediated through gain of function of immune effector cells, namely natural killer cells [174]. In lymphoma cells, exposure to lenalidomide leads to cereblon-mediated degradation of transcription factors Ikaros (IKZF1), Aiolos (IKZF3) and interferon response 4 (IRF4) and subsequent inhibition of nuclear factor kappa B [175]. Despite the well-known direct effect, the principal anti-tumor efficacy of lenalidomide appears to be based on enhanced NK cell-mediated cytotoxicity against lymphoma cells via increasing NK cell numbers and fostering their activation, and proliferation (Figure 1F). The lenalidomide-induced immunomodulation of NK cells results in increased lytic immunological synapse formation and eradication of the malignant target cells via perforin/granzyme B pathway [174,176]. Single-agent lenalidomide has been approved for the therapy of R/R MCL based on the repeatedly demonstrated single-agent activity [177,178].

#### 6.1.1. Lenalidomide and Anti-CD20 Antibodies: R^2^ and GALEN Regimen

The lenalidomide-mediated enhancement of NK cell functions results in anti-tumor synergy with therapeutic monoclonal antibodies, whose mode-of-action is based predominantly on the antibody-dependent cell-mediated cytotoxicity (ADCC). In the landmark phase 3 AUGMENT trial, median progression-free survival for lenalidomide plus rituximab (R^2^ regimen) versus placebo plus rituximab was 39.4 months and 14.1 months, respectively [179]. Another phase 3 clinical study evaluating the efficacy of lenalidomide and rituximab combination in indolent lymphomas is a MAGNIFY trial. The interim analysis of the MAGNIFY trial (Clinical trial identifier NCT01996865) presented at the American Society of Clinical Oncology annual meeting in 2019 demonstrated tolerable safety profile and promising anti-lymphoma activity in patients with R/R FL and MZL. Median PFS was 30.2 months and 38.4 months in patients with R/R FL and R/R MZL. Based on the results of these two key clinical trials (AUGMENT and MAGNIFY), lenalidomide in combination with rituximab was approved in 2019 by the Food and Drug Administration for the therapy of R/R FL and R/R MZL. In the front-line settings, the clinical trial RELEVANCE did not demonstrate difference among patients treated with the combination of lenalidomide and rituximab and patients treated with the combination of chemotherapy and rituximab [180]. Outside indolent lymphomas, lenalidomide and rituximab (R^2^ regimen) demonstrated promising anti-lymphoma activity in patients with mantle cell lymphoma, diffuse large B-cell lymphoma and primary CNS lymphoma [181,182,183,184,185,186,187]. The success of the R^2^ regimen across B-NHL subtypes was a rationale for design and clinical testing of another promising combination of lenalidomide and obinutuzumab, a glycoengineered antibody with enhanced ADCC. A clinical trial of obinutuzumab and lenalidomide (GALEN) in R/R follicular lymphoma (NCT01582776) demonstrated that the GALEN regimen was active in previously-treated patients with manageable toxicity [188]. In addition to indolent lymphomas, the GALEN regimen was active also in aggressive lymphomas, especially in the ABC-DLBCL subtype [189].

#### 6.1.2. Lenalidomide as a Maintenance Therapy

Besides its widespread used in various induction and salvage regimen, lenalidomide demonstrated single-agent activity as a maintenance therapy. Lenalidomide maintenance after R-CHOP-based induction in patients with newly diagnosed DLBCL was associated with prolonged progression-free survival, but unchanged overall survival [190]. In addition, the toxicity was significant in the lenalidomide maintenance arm. Recent data reported activity of lenalidomide maintenance in primary CNS lymphoma [191,192]. Ongoing clinical trials that incorporate lenalidomide in experimental therapy of NHL are displayed in Table 9.

### 6.2. Avadomide

Avadomide is a second-generation cereblon-modulating IMiD. Avadomide monotherapy was well-tolerated and demonstrated clinical efficacy in R/R DLBCL patients resulting in prolonged PFS in the patients, whose tumors were enriched with immune cells [193]. Avadomide is currently tested in combination with obinutuzumab and with R-CHOP in patients with R/R DLBCL [194].

## 7. Toxicity Associated with Immunotherapy

Immunotherapy has distinct toxicities, some of which are class-dependent; however, some of them may be expected through different classes of immunotherapy agents. We will shortly review infusion reactions to monoclonal antibodies, checkpoint inhibitors immune mediated adverse effects, cytokine release syndrome and neurotoxicity.

### 7.1. Infusion Reactions

Infusion reactions to antibodies were first described in rituximab and trastuzumab, as combinations of chills, sweating, fever, rash, pruritus, blood pressure changes, tachycardia, shortness of breath, angioedema, and bronchospasm [195,196]. Infusion reactions occur most frequently during the first cycle of therapy and their frequency is both drug and disease dependent. High tumor burden, especially presence of malignant cells in the circulation, correlated with more severe reactions, and in one report, occurrence of infusion reaction positively correlated with treatment outcome in NHL [197]. Most infusion reactions can be classified as a “classical type” cytokine release syndrome [198], though IgE-mediated anaphylactic reactions and immunocomplex-mediated serum sickness-like reactions may occur in the minority of patients [199]. Infusion reactions can be prevented by application of glucocorticoids, acetaminophen, and antihistamines, as well as by slow, gradually increasing rate of infusion administered for the first time [200]. Division of the first dose in two consecutive days or dose escalation can be appropriate in clinical situations with high risk of infusion reaction [13,201]. In the minority of patients where severe infusion reactions persist in subsequent treatment cycles, desensitization protocol may be used [202].

### 7.2. Immune-Mediated Adverse Events Associated with Checkpoint Inhibitors

Adverse events associated with immune checkpoint inhibitors are caused by non-specific deregulation of T-lymphocyte function by anti-CTLA-4, anti-PD-1, or anti-PD-L1 blockade. Most frequently affected organs include skin, gastrointestinal tract, and lungs, while endocrine, musculoskeletal, renal, nervous, hematologic, cardiovascular, and ocular systems may be also involved [203]. These side effects occur in up to 90% of patients after CTLA-4 blockade and up to 70% of patients after PD-1/PD-L1 blockade [67,204]. Though most of these adverse events occur in 3–6 months after initiation of therapy, delayed toxicities up to 1 year after the first dose may be observed. As there are no established diagnostic tests, diagnosis is usually made by exclusion of other possible causes of organ involvement. Comprehensive guidelines for organ toxicity management associated with checkpoint inhibitor therapy are available [205,206]. In general, immune checkpoint inhibitors may be continued in most cases of grade 1 organ toxicities, except for some neurologic, hematologic and cardiac side-effects. Temporary discontinuation of treatment with low dose steroids (0.5–1 mg/kg prednisolone) in grade 2, or high-dose steroids (1–2 mg/kg prednisolone) in grade 3 toxicity is recommended. Treatment is usually terminated with grade 4 toxicity.

### 7.3. Cytokine Release Syndrome

Cytokine release syndrome (CRS) is a combination of fever, hypoxia, hypotension, and capillary leak syndrome, with or without other organ manifestations (renal failure, coagulopathy, or neurological symptoms) [198]. Laboratory finding include elevated levels of CRP, IL-1β, IL-2, IL-6, IL-10, IFN-γ, and TNF-α [207]. Other laboratory features, such as elevated ferritin and triglyceride levels, overlap with hemophagocytic histiocytosis-macrophage activation syndrome (HLH-MAS) [208]. While CRS induced by classical monoclonal antibodies usually starts during the antibody administration and abates after its interruption, CRS associated with CAR T-cell therapy or bsAbs is characterized by later onset, usually 1 to 3 days after therapy initiation, but it may occur as late as after two weeks. As with classical mAbs, frequency and severity of CRS after CAR T-cells or bsAbs is dependent both on the product and the treated disease. On a pathophysiological level, CRS seems to be mediated by T-lymphocyte and myeloid cell interactions [209]. Upon contact with the tumor cells, T-cells secrete inflammatory cytokines such as TNF-α and IFN-γ, which in turn stimulate secretion of IL-1, IL-6, inducible nitric oxide synthase (iNOS) and other cytokines by monocytes and macrophages. These cytokines are then responsible for clinical symptoms, including fever and capillary leak syndrome, which leads to hypotension, pulmonary edema, and hypoxia. Direct contact of T-cells and myeloid cells (e.g., via CD40-CD40L ligation) may be also involved [210]. Systemic CRS may be preceded by localized CRS, which manifests as redness, swelling, and enlargement in place of tumor involved lymph nodes [211]. Several grading systems for CRS were employed to quantify this side-effect in diverse clinical trials and for therapy guidance, namely Lee, Penn, Memorial Sloan-Kettering Cancer Center (MSKCC) and CARTOX [144,145,212,213]. Management of CRS is both symptomatic and causal. For grade 1 CRS, antipyretics and non-steroidal antirheumatic drugs are sufficient. When hypotension occurs, early use of vasopressors is strongly recommended, as capillary leak may lead to pulmonary edema during high-volume fluid resuscitation. In patients with fully developed pulmonary edema, oxygenotherapy with positive airway pressure should be used. Anti-IL-6 antibody tocilizumab is currently a mainstay of causal therapy of grade 3–4 CRS. Low-dose dexamethasone is used in non-responders [214]. Patients refractory to tocilizumab and dexamethasone remain difficult to treat, though several promising agents are being evaluated in ongoing clinical trials, including IL-1 receptor antagonist anakinra [215], JAK/STAT pathway inhibitor itacinib [216], and tyrosine kinase inhibitor ibrutinib [155].

### 7.4. Neurotoxicity

Neurotoxicity represents another specific adverse event associated with T-cell redirecting therapies. Neurotoxicity, or the immune effector cell-associated neurotoxicity syndrome (ICANS) may have distinct clinical manifestations including confusion, aphasia, apraxia, frontal release sings, meningism, palsy, seizures, cerebral edema, or coma. Expressive aphasia, especially the inability to name objects, is an early and quite specific symptom [217]. Various neurological symptoms were also described as part of the classical CRS, but currently, ICANS is a distinct clinical entity because of different temporal presentation and inefficacy of anti-IL-6 directed therapy. In anti-CD19 CAR T-cell-based trials, the incidence of ICANS ranges from 20% to 64%, with grade ≥ 3 toxicity occurring in 11–42%. In the trials with blinatumomab, grade ≥ 3 ICANS occurred in 22–24% of patients [47,49]. The median time to onset of ICANS in different clinical studies was 4–5 days since the infusion administration, with median duration of 5–12 days [218,219]. Risk factors for ICANS include high tumor burden, preexisting neurologic conditions, higher peak of CAR T-lymphocyte count and early and/or severe CRS. Most of the ICANS symptoms resolve within a few weeks, but irreversible or even fatal toxicities have been reported [220]. The pathogenesis of ICANS is not clearly understood, but similarly to CRS, it is believed to be induced by the inflammatory cascade triggered by activation of T-lymphocyte and their interaction with myeloid cells. Endothelial dysfunction induced by the inflammatory cascade is also believed to play an important role in ICANS [221]. Elevated levels of a variety of cytokines, such as IL-1 and IL-6, IL-8, IL-10, TNFα, IFNγ, MCP-1 or ferritin, were reported in the plasma and cerebrospinal fluid (CSF) of patients with ICANS. High-grade neurotoxicity was associated with increased levels of angiotensin II, von Willebrand factor (vWF) and IL-8 (all stored in Weibel-Palade bodies in endothelial cells), and with clinical manifestation of disseminated intravascular coagulation. The histopathological examination proved dysfunction of blood-brain barrier, activation of microglia in some cases and multifocal microthrombi and microhemorrhages in CNS. American Society for Transplantation and Cellular Therapy (ASTCT) ICANS consensus grading systems is based on the level of consciousness, presence of seizures, motoric deficits, signs and symptoms of elevated intracranial pressure, and simple neurological assessment known as immune-effector cell associated encephalopathy (ICE) score. ICE score evaluates attention, orientation, and abilities of writing, naming objects, and following commands. Treatment of ICANS is based on corticosteroids together with symptomatic approach, such as anti-seizure medication and airway protection. IL-1 receptor antagonist anakinra was successfully used in patients with severe ICANS [215] and prospective studies evaluating this agent are ongoing (NCT04432506).

## 8. Conclusions

Without any doubt, immunotherapy has radically changed our possibility to fight lymphomas including chemotherapy-refractory cases (Table 10).

It is evident that immunotherapy alone can induce durable remissions with a potential cure for many patients with otherwise resistant disease. Though immunotherapy may be effective even in late stages of disease, in these situations its efficacy may be compromised by patients’ poor performance status, destruction and exhaustion of cellular and humoral components of the immune system by previous lines of immunosuppressive therapies, and biologically highly aggressive disease. The use of immunotherapy at earlier clinical phases (or even in the front-line setting) might thus lead to increased response rates, better tolerability, and longer remissions. In the upcoming years, next-generation biologicals with better pharmacokinetic properties and improved safety profiles will further extend the indication of immunotherapy to even older and more comorbid patients. Since the clinical usage of allogeneic transplantation, it has become evident that the most effective setting for immunotherapy is eradication (or at least control) of minimal residual disease, while immunotherapy approaches appear less-effective in eliminating bulky tumor masses. Published data support this observation across diverse lymphoma subtypes and various other immunotherapy modalities. One of the most relevant pretreatment factors of CAR19 T-cells efficacy in DLBCL is tumor bulk. While PD-1 inhibition was largely ineffective as a salvage therapy in DLBCL patients, its administration as a maintenance after HDT-ASCT consolidation had clinical benefit. Anti-leukemic activity of the bispecific BiTE blinatumomab decreases with increasing pretreatment leukemic cell load. Use of immunotherapy in consolidative or maintenance setting, after prior debulking with chemotherapy or targeted agents and subsequent immunotherapy-mediated eradication of the residual lymphoma clone, may thus represent a feasible future approach to patients with newly diagnosed lymphoma. In follicular lymphoma or mantle cell lymphomas, where maintenance therapy had demonstrated large clinical benefit already with CD20 antibody rituximab, the innovative immunotherapy approaches might offer far better control of the residual lymphoma clone. Which immunotherapy modality will prevail in particular lymphoma subtypes will depend on the results of the currently ongoing clinical trials. It is also plausible that different immunotherapy agents will be used in combination(s) with each other, inducing synthetic lethality toward lymphoma cells.

In conclusion, immunotherapy offers a curative perspective both to patients with NHL subtypes currently considered incurable and to those with curable diagnoses after the failure of currently used standard-of-care therapies. For the first time in history, cure for most patients with non-Hodgkin lymphomas seems to be an achievable goal.

## Figures and Tables

**Table 1:** Selected clinical trials that incorporate blinatumomab in experimental therapy of B-NHL.

Drug Combination	Target Antigens	Mode of Action of the Combination Agent(s) Other Than Bispecific Antibody	Study Phase	Disease Status	Estimated Study Completion Date	ClinicalTrials.gov Identifier (Other Identifier)
blinatumomab	CD19/CD3		2	R/R indolent B-NHL	December 2023	NCT02811679
blinatumomab	CD19/CD3		1	R/R indolent B-NHL	May 2022	NCT02961881
blinatumomab	CD19/CD3		1	DLBCL after ASCT	December 2023	NCT03072771
blinatumomab + lenalidomide	CD19/CD3	immunomodulatory agent lenalidomide	1	R/R B-NHL	December 2020	NCT02568553
blinatumomab + pembrolizumab	CD19/CD3	immune check-point PD-1 inhibitor pembrolizumab	1	R/R DLBCL	January 2026	NCT03340766 (KEYNOTE-348)

Abbreviations: ASCT= autologous stem cell transplantation; DLBCL= diffuse large B-cell lymphoma; PD-1= programmed death 1; R/R= relapsed/refractory; B-NHL= B cell non-Hodgkin lymphomas.

**Table 2:** Selected clinical trials that incorporate mosunetuzumab in experimental therapy of B-NHL.

Drug Combination	Target Antigens	Mode of Action of the Combination Agent(s) Other Than Bispecific Antibody	Study Phase	Disease Status	Estimated Study Completion Date	ClinicalTrials.gov Identifier (Other Identifier)
Mosunetuzumab ± atezolizumab	CD20/CD3	immune check-point PD-L1 inhibitor atezolizumab	1	R/R B-NHL and CLL	October 2021	NCT02500407
Mosunetuzumab + polatuzumab vedotin compared to bendamustine + rituxumab + polatuzumab vedotin	CD20/CD3	anti-CD79B antibody-drug conjugate polatuzumab-vedotin, anti-CD20 rituximab, new cytostatic agent bendamustine	1B/2	R/R DLBCL and R/R FL	June 2022	NCT03671018
Mosunetuzumab + lenalidomide, glofitamab + lenalidomide or glofitamab + lenalidomide + obinutuzumab	CD20/CD3	immunomodulatory agent lenalidomide, glycoengeneered anti-CD20 mAb obinutuzumab	1	newly dg DLBCL, R/R DLBCL and R/R FL	August 2022	NCT04246086
Mosunetuzumab + CHOP or mosunetuzumab + polatuzumab vedotin + CHP	CD20/CD3	chemotherapy CHOP, anti-CD79B antibody drug conjugate polatuzumab-vedotin	1B/2	newly dg DLBCL, R/R B-NHL	June 2022	NCT03677141
Mosunetuzumab	CD20/CD3		1B/2	newly dg DLBCL	April 2023	NCT03677154
Mosunetuzumab or Glofitamab + GemOx	CD20/CD3	chemotherapy gemcitabine and oxaliplatin (GemOx)	1	R/R DLBCL	March 2021	NCT04313608

Abbreviations: CHOP= cyclophosphamide + doxorubicin + vincristine + prednisone; CLL= chronic lymphocytic leukemia; DLBCL= diffuse large B-cell lymphoma; FL= follicular lymphoma; PD-L1= programmed death ligand 1; R/R= relapsed/refractory; B-NHL= B cell non-Hodgkin lymphomas.

**Table 3:** Selected clinical trials that incorporate glofitamab in experimental therapy of B-NHL.

Drug Combination	Target Antigens	Mode of Action of the Combination Agent(s) Other Than Bispecific Antibody	Study Phase	Disease Status	Estimated Study Completion Date	ClinicalTrials.gov Identifier (Other Identifier)
Glofitamab + GemOx compared to rituximab + GemOx	CD20/CD3	cytostatics gemcitabine and oxaliplatin (GemOx), anti-CD20 rituximab	3	R/R DLBCL	March 2022	NCT04408638
Glofitamab + obintuzumab with obinutuzumab pretreatment	CD20/CD3	glycoengeneered anti-CD20 obinutuzumab	1	R/R B-NHL	June 2022	NCT03075696
Glofitamab + obintuzumab or rituximab + CHOP with obinutuzumab pretreatment	CD20/CD3	chemotherapy CHOP, anti-CD20 obintuzumab, anti-CD20 rituximab	1	newly dg and R/R B-NHL	December 2023	NCT03467373
Glofitamab + atezolizumab or polatuzumab-vedotin with obinutuzumab pretreatment	CD20/CD3	PD-L1 inhibitor atezolizumab, anti-CD79B antibody-drug conjugate polatuzumab-vedotin	1	R/R B-NHL	August 2021	NCT03533283
Glofitamab + RO7227166 with obinutuzumab pretreatment	CD20/CD3	CD19 Targeted 4-1BB Ligand RO7227166	1	R/R B-NHL	January 2023	NCT04077723
Glofitamab + lenalidomide +/− obinutuzumab	CD20/CD3	Immunomodulatory agent lenalidomide	1	R/R FL	August 2022	NCT04246086

Abbreviations: CHOP= cyclophosphamide + doxorubicin + vincristine + prednisone; DLBCL= diffuse large B-cell lymphoma; FL= follicular lymphoma; PD-L1= programmed death ligand 1; R/R= relapsed/refractory; B-NHL= B cell non-Hodgkin lymphomas.

**Table 4:** Selected clinical trials that incorporate bispecific antibodies in experimental therapy of NHL.

Drug Combination	Target Antigens	Mode of Action of the Combination Agent(s) Other Than Bispecific Antibody	Study Phase	Disease Status	Estimated Study Completion Date	ClinicalTrials.gov Identifier (Other Identifier)
REGN1979 (odronextamab)	CD20/CD3		1	R/R B-NHL	August 2026	NCT03888105
REGN1979 (odronextamab)	CD20/CD3		1	R/R B-NHL and CLL	April 2025	NCT02290951
AFM13 + NK cells	CD30/CD16A	modified umbilical cord blood immune cells (natural killer [NK] cells)	1	R/R Hodgkin and CD30+ B-NHL	April 2023	NCT04074746
AFM13	CD30/CD16A		2	R/R T-NHL and tMF	February 2023	NCT04101331
JNJ-75348780	CD22/CD3		1	R/R MCL	May 2023	NCT04540796
TG-1801 + ublituximab	CD19/CD47	chimeric anti-CD20 mAb	1	R/R B-NHL	August 2021	NCT03804996
RO7227166 + obinutuzumab + glofitamab	CD19/4-1BB	glycoengineered anti-CD20 antibody obinutuzumab, anti-CD3/CD20 bispecific antibody glofitamab	1	R/R B-NHL	January 2023	NCT04077723

Abbreviations: CLL= chronic lymphocytic leukemia; R/R= relapsed/refractory; B-NHL= B cell non-Hodgkin lymphomas; MCL= mantle cell lymphoma; tMF= transformed mycosis fungoides.

**Table 5:** Selected checkpoint inhibitors currently being evaluated in patients with NHL.

Name	Trade Name	Developed by	Structure	Target	First Approval by US FDA for the Treatment of Cancer	Number of Studies in Patients with NHL Registered at ClinicalTrials.gov
Ipilimumab	Yervoy	Bristol-Myers-Squibb	human IgG1	CTLA-4	2011	13
Tremelimumab	N/A	AstraZeneca	human IgG2	CTLA-4	N/A	3
Pembrolizumab	Keytruda	Merck	humanized IgG4	PD-1	2014	60
Nivolumab	Opdivo	Bristol-Myers-Squibb	human IgG4	PD-1	2014	41
Pidilizumab	N/A	Medivation	human IgG1	PD-1	N/A	3
Durvalumab	Imfinzi	AstraZeneca	human IgG1	PD-L1	2020	19
Avelumab	Bavencio	Merck, Pfizer	human IgG1	PD-L1	2017	9
Atezolizumab	Tenetriq	Roche	humanized IgG1	PD-L1	2016	20

N/A—not applicable.

**Table 6:** Selected clinical trials that incorporate immune checkpoint inhibitors in experimental therapy of NHL.

Drug Combination	Mode of Action of the Combination Agent(s) Other Than Immune Checkpoint Inhibitors	Study Phase	Disease Status	Estimated Study Completion Date	ClinicalTrials.gov Identifier (Other Identifier)
Nivolumab + R(ituximab)-GemOx compared to R-GemOx	immunochemotherapy gemcitabine + oxaliplatin (GemOx)	2/3	R/R elderly B-NHL	November 2024	NCT03366272 (NIVEAU)
Avelumab +/− Utomilumab +/− Rituximab +/− Azacitidine +/− bendamustin +/− Gemcitabine +/− Oxaliplatine	CD137 (4-1BB) antigen agonist antibody utomilumab, anti-CD20 antibody rituximab, epigenetic modulator azacitidine, conventinal chemotherapy GemOx	1/3	R/R DLBCL	December 2019	NCT02951156 (JAVELIN DLBCL)
Nivolumab + DA-EPOCH-R + Nivolumab as a consolidation	immunochemotherapy regimen (dose-adjusted EPOCH-R)	2	B-NHL	December 2021	NCT03749018
Nivolumab + Copanlisib	pan-PI3K inhibitor copanlisib	2	R/R DLBCL, PMBCL	October 2021	NCT03484819
Pembrolizumab		2	untreated B-NHL	September 2024	NCT03498612
Pembrolizumab		2	R/R grey-zone lymphoma, R/R PCNSL, R/R DLBCL	July 2022	NCT03255018
Pembrolizumab + R-CHOP	R-CHOP immunochemotherapy regimen	2	DLBCL, high-grade B-NHL	August 2024	NCT03995147
Pembrolizumab + Rituximab +/− Lenalidomide	anti-CD20 antibody, immunomodulatory agent lenalidomide	2	R/R FL, R/R DLBCL	November 2021	NCT02446457
Durvalumab + R-CHOP/R2-CHOP	standard immunochemotherapy, immunomodulatory agent lenalidomide	2	DLBCL	March 2023	NCT03003520
Ipilimumab + Lenalidomide	immunomodulatory agent lenalidomide	2	NHL (post-HSCT)	June 2021	NCT01919619
Pembrolizumab + ALX-148	CD47 antagonist ALX-148	1	NHL, solid tumors	December 2021	NCT03013218

Abbreviations: B-NHL= B cell non-Hodgkin lymphomas; CHOP= cyclophosphamide + doxorubicin + vincristine + prednisone; DLBCL= diffuse large B-cell lymphoma; EPOCH-R= etoposide, prednisone, vincristine, cyclophosphamide, doxorubicin, rituximab; FL= follicular lymphoma; HSCT= hematopoietic stem cell transplantation; PCNS= primary CNS lymphoma; PMBCL= primary mediastinal B-cell lymphoma; R/R= relapsed/refractory.

**Table 7:** Characterization of clinically approved CAR T-cell products.

Full Generic Name	Axicabtagene Ciloleucel	Tisagenlecleucel	Lisocabtagene Maraleucel
Shortened name	Axi-cel	Tisa-cel	Liso-cel
Manufacturer	Kite-Gilead	Novartis	Bristol-Myers Squibb
Registration study	ZUMA-1	JULIETT	TRANSCEND
	Number of patients		
Total	111	165	344
Infused	101 (91% of total)	111 (67% of total)	269 (78% of total)
DLBCL *de novo*	77 (76%)	88 (79%)	137 (51%)
DLBCL transformed	16 (16%)	21 (19%)	78 (29%)
PMBL	8 (8%)	0 (0%)	15 (6%)
Double/triple hit	NR	19 (27%) *	36 (13%)
Other	0 (0%)	2 (2%) **	3 (1%) #
	Age		
Median (range)	58 (23–76)	56 (22–76)	63 (54–70)
≥65 years	24 (24%)	25 (23%)	112 (42%)
	Gender		
Male	68 (67%)	NR	174 (65%)
Female	33 (33%)	NR	95 (35%)
	Disease status		
Primary refractory	2 (2%)	NR	NR
Relapse after ASCT	21 (21%)	54 (49%)	94 (35%)
≥3 lines of therapy	70 (69%)	57 (52%)	139 (52%)
Bridging therapy administered	0	92%	159 (59%)
	Response rate		
ORR	83%	52%	73%
CR	58%	40%	53%
	Survival		
OS	52% at 18 months	Median, 12 months	Median, 21.1 months
PFS	Median, 5.9 months	Median, 2.9 months	Median, 6.8 months
DOR	Median, 11.1 months	65% at 12 months	55% at 12 months
	Adverse events		
	Cytokine release syndrome		
All grades	93%	64 (58%)	113 (42%)
Grade 3–4	13%	24 (22%)	6 (2%)
	Neurotoxicity		
All grades	34%	23 (21%)	80 (30%)
Grade 3–4	21%	13 (12%)	27 (10%)
	Infections		
All grades	NR	38 (34%)	not reported
Grade 3–4	NR	22 (20%)	not reported

Abbreviations: ASCT = autologous stem cell transplantation; DOR = duration of response; DLBCL = diffuse large B-cell lymphoma; CR = complete remission; ORR = overall response rate; OS = overall survival; PFS = progression-free survival; PMBCL = primary mediastinal B-cell lymphoma; NR = not reported; * of 70 examined patients; ** Not specified; # Follicular lymphoma grade 3.

**Table 8:** Selected clinical trials that incorporate CAR T-cell products in experimental therapy of NHL.

Drug Combination	Mode of Action	Study Phase	Disease Status	Estimated Study Completion Date	ClinicalTrials.gov Identifier (Other Identifier)
Axi-cel	Anti-CD19 CAR T-cells versus ASCT (2nd line therapy)	3	R/R hgB-NHL	January 2022	NCT03391466 (ZUMA-7)
Liso-cel	Anti-CD19 CAR T-cells versus ASCT (2nd line therapy)	3	R/R hgB-NHL	January 2024	NCT03575351 (TRANSFORM)
Tisa-cel	Anti-CD19 CAR T-cells versus ASCT (2nd line therapy)	3	R/R hgB-NHL	December 2025	NCT03570892 (BELINDA)
Axi-cel	Anti-CD19 CAR T-cells	2	R/R FL, R/R MZL	February 2022	NCT03105336 (ZUMA-5)
Liso-cel	Anti-CD19 CAR T-cells	2	R/R B-NHL ineligible for ASCT	April 2021	NCT03483103 (TRANSCEND-PILOT-017006)
KTE-X19	Anti-CD19 CAR T-cells	1	R/R SLL/CLL	August 2021	NCT03624036
Liso-cel + ibrutinib	Anti-CD19 CAR T-cells + BTK inhibitor ibrutinib	1/2	R/R CLL/SLL	October 2021	NCT03331198
Axi-cel + acalabrutinib	BTK inhibitor acalabrutinib administered before leukapheresis	1/2	R/R hgB-NHL	March 2024	NCT04257578
CD30.CAR T cells	Anti-CD30 CAR T-cells	1	R/R HL, CD30+ NHL	April 2021	NCT02917083 (RELY-30)
AUTO4	Anti-TRBC1 CAR T-cells	1/2	R/R T-NHL	July 2021	NCT03590574
CD4CAR	Anti-CD4 CAR T-cells	1	R/R T-NHL	December 2022	NCT03829540
Axi-cel	Anti-CD19 CAR T-cells	1	DLBCL (PET+ after 2 cycles of therapy)	June 2021	NCT03761056 (ZUMA-12)
ALTCAR.CD30	ASCT followed by anti-CD30 CAR T-cells	1	R/R HL, CD30+ NHL	September 2021	NCT02663297
AlloSCT + CAR-T	T-cell depleted alloSCT + donor anti-CD19 CAR T-cell-based consolidation	1	B-ALL, CLL, NHL	September 2023	NCT04556266
CAR 20/19	Bispecific anti-CD20/anti-CD19 CAR T-cells	1/2	R/R B-NHL	May 2023	NCT04186520
Liso-cel + avadomide, iberdomide, ibrutinib, or durvalumab	Anti-CD19 CAR T-cells in combination with immunomodulatory drugs avadomide/iberdomide, BTK inhibitor ibrutinib or anti PD-L1 checkpoint durvalumab	1/2	R/R hgB-NHL	August 2023	NCT03310619 (PLATFORM)
AUTO3 + pembrolizumab	Dual anti-CD19/anti-CD22 CAR T-cells + anti PD-1 immune checkpoint inhibitor pembrolizumab	1/2	R/R hgB-NHL	March 2021	NCT03287817 (ALEXANDER)
CD19-PD1-CART	Anti-CD19 CAR T-cells with PD-1/CD28 co-stimulation	1	R/R B-NHL	July 2021	NCT04163302
CD7.CAR	Anti-CD7 CAR T-cells with CD7 deletion	1	R/R CD7+ T-cell malignancies	May 2023	NCT03690011 (CRIMSON)
iC9/CAR.19/IL15-Transduced CB-NK Cells	Cord blood-derived allogeneic anti-CD19 NK-cells with IL-15 and inducible caspase 9	1/2	B-ALL, CLL, B-NHL	June 2022	NCT03056339
CD19.CAR-aNKT	Anti-CD19 allo CAR NK/T cells with IL-15	1	R/R ALL, CLL, hgB-NHL	April 2023	NCT03774654

Abbreviations: alloSCT= allogeneic stem cell transplantation; ASCT= autologous stem cell transplantation; B-ALL= B-cell acute lymphoblastic leukemia; BTK= Bruton tyrosine-kinase inhibitor; CLL/SLL= chronic lymphocytic leukemia/small lymphocytic lymphoma; DLBCL= diffuse large B-cell lymphoma; FL= follicular lymphoma; hgB-NHL= high-grade B-NHL; HL= Hodgkin lymphoma; HSCT= hematopoietic stem cell transplantation; iC9= inducible caspase 9; MZL= marginal zone lymphoma; R/R= relapsed/refractory; NHL= non-Hodgkin lymphomas.

**Table 9:** Selected clinical studies in NHL that incorporate lenalidomide.

Drug Combination	Mode of Action of the Drug Combination	Study Phase	Target Population	Estimated Study Completion Date	GovTrial Denominator
Brentuximab vedotin + lenalidomide + rituximab	anti-CD30 antibody-drug conjugate brentuximab-vedotin, anti-CD20 rituximab	3	R/R DLBCL	December 2025	NCT04404283
Lenalidomide + R-ICE	anti-CD20 rituximab, chemotherapy ifosfamide, carboplatin, etoposide (ICE)	1/2	R/R DLBCL	December 2025	NCT02628405
lenalidomide + R-CHOP	chemotherapy regimen R-CHOP	2	DLBCL (CNS prophylaxis)	October 2024	NCT04544059
Lenalidomide + R-GemOx	anti-CD20 rituximab, gemcitabine, oxaliplatin	1	Newly dg. elderly DLBCL	December 2024	NCT04432402
Lenalidomide + Rituximab + Dexamethasone	anti-CD20 rituximab	2	Newly dg. elderly DLBCL	March 2021	NCT02955823
Axicabtagene Ciloleucel + rituximab or lenalidomide	anti-CD20 rituximab	1	R/R DLBCL	September 2036	NCT04002401 (Zuma-14)
Brentuximab vedotin + lenalidomide + rituximab	anti-CD30 antibody-drug conjugate brentuximab-vedotin, anti-CD20 rituximab	3	R/R DLBCL	December 2025	NCT04404283
lenalidomide + obinutuzumab + venetoclax	anti-CD20 obinutuzumab, BCL2 inhibitor venetoclax	1/2	Newly dg. FL	November 2026	NCT03980171 (LEVERAGE)
Lenalidomide + Acalabrutinib + Rituximab	BTK inhibitor acalabrutinib, anti-CD20 rituximab	2	Newly dg. FL	March 2025	NCT04404088
Venetoclax, Lenalidomide and Rituximab	BCL2 inhibitor venetoclax, anti-CD20 antibody rituximab	1	Newly dg. MCL	July 2022	NCT03523975
Lenalidomide + Acalabrutinib + Rituximab	BTK inhibitor acalabrutinib, anti-CD20 rituximab	2	Newly dg. MCL	November 2024	NCT03863184
Venetoclax, Lenalidomide and Rituximab	BCL2 inhibitor venetoclax, anti-CD20 antibody rituximab	1/2	R/R MCL	June 2022	NCT03505944 (VALERIA)
Carfilzomib + Lenalidomide + Dexamethasone	Irreversible proteasome inhibitor carfilzomib	1	R/R MCL (ibrutinib refractory)	April 2022	NCT03891355 (FIL_KLIMT)
Lenalidomide maintenance		2	R/R T-NHL (after salvage therapy)	November 2023	NCT03730740 (Lemon-T)
Lenalidomide + Brentuximab Vedotin	anti-CD30 antibody-drug conjugate brentuximab-vedotin	1	R/R PTCL	August 2021	NCT03302728
Lenalidomide + Ibrutinib + Rituximab	BTK inhibitor ibrutinib, anti-CD20 rituximab	1	primary/secondary CNS lymphoma	November 2021	NCT03703167

Abbreviations: BCL2= B-cell lymphoma; B-NHL= B cell non-Hodgkin lymphomas; BTK= Bruton tyrosine-kinase; MCL= mantle cell lymphoma; GemOx= gemcitabine and oxaliplatin; R/R= relapsed/refractory.

**Table 10:** Selected immunotherapy approaches clinically approved for treatment of NHL.

Class of Agents/Agent	Targeted Structure	Effective in NHL Subtypes
Monospecific monoclonal antibodies		
Rituximab	CD20	All B-NHL [11,12]
Obinutuzumab	CD20	CLL/SLL [13], FL frontline [14], R/R FL [15]
Tafasitamab	CD19	R/R B-NHL [26], R/R DLBCL [27]
Alemtuzumab	CD52	Mycosis fungoides [33], T-PLL [34]
Mogamulizumab	CCR4	Adult T-cel leukemia/lymphoma [37,38]
Bispecific monoclonal antibodies		
Blinatumomab	CD3-CD19	R/R B-NHL [48] R/R DLBCL [49,50]
Mosunetuzumab	CD3-CD20	R/R B-NHL [52]
Glofitamab	CD3-CD20	R/R B-NHL [56,57]
Checkpoint inhibitors		
Pembrolizumab	PD-1	R/R PMBCL [80,81], Richter’s syndrome [93], mycosis fungoides [95]
Nivolumab	PD-1	R/R PMBCL [83], PCNSL and PTL [77]
Pidilizumab	PD-1	DLBCL after autologous SCT [75]
CAR-T cells		
Tisagenlecleucel	CD19	R/R aggressive NHL [141]
Axicabtagene ciloleucel	CD19	R/R aggressive NHL [142,143]
Lisocabtagene maraleucel	CD19	R/R aggressive NHL [144]
Brexucabtagene autoleucel	CD19	R/R MCL [150]
Immunomodulatory agents		
Lenalidomide	Cereblon	R/R FL and MZL [180] MCL frontline [182,183], R/R PCNSL [184], R/R DLBCL [185]
Avadomide	Cereblon	R/R DLBCL [194]

Abbreviations: B-NHL = B-non Hodgkin’s lymphoma, CLL/SLL = chronic lymphocytic leukemia/small lymphocytic lymphoma, FL = follicular lymphoma, R/R = relapsed/refractory, DLBCL = diffuse large B-cell lymphoma, T-PLL = T-prolymphocytic leukemia, PMBCL = primary mediastinal B-cell lymphoma, PCNSL = primary central nervous system lymphoma, PTL = primary testicular lymphoma, SCT = stem cell transplantation, MCL = mantle cell lymphoma, MZL = marginal zone lymphoma.
